# MRI assessment of hepatocellular carcinoma after locoregional therapy

**DOI:** 10.1186/s13244-019-0690-1

**Published:** 2019-01-29

**Authors:** Rasha S. Hussein, Wahid Tantawy, Yasser A. Abbas

**Affiliations:** Radiology Department, Faculty of Medicine, Ain Shams University and MR Unit of Misr Radiology Center, Cairo, Egypt

**Keywords:** Hepatocellular carcinoma, Locoregional treatment, Magnetic resonance imaging, Treatment response of HCC, LI-RADS v2018

## Abstract

Liver cirrhosis and hepatocellular carcinoma (HCC) constitute one of the major causes of morbidity, mortality, and high health care costs worldwide. Multiple treatment options are available for HCC depending on the clinical status of the patient, size and location of the tumor, and available techniques and expertise. Locoregional treatment options are multiple. The most challenging part is how to assess the treatment response by different imaging modalities, but our scope will be assessing the response to locoregional therapy for HCC by MRI. This will be addressed by conventional MR methods using LI-RADS v2018 and by functional MR using diffusion-weighted imaging, perfusion, and highlighting the value of the novel intravoxel incoherent motion (IVIM).

## Key points

- To know the available locoregional therapies for HCC.
- To compare various criteria for Response Assessment in HCC.
- To highlight MRI criteria for LI-RADS v2018 in treatment response assessment for each locoregional therapy.
- To demonstrate some pitfalls in assessment after various available locoregional therapy and the available functional methods for treatment response assessment.
- To highlight the novel intravoxel incoherent motion (IVIM) which could be used as quantitative tool.

## Introduction

**Fig. 1:**
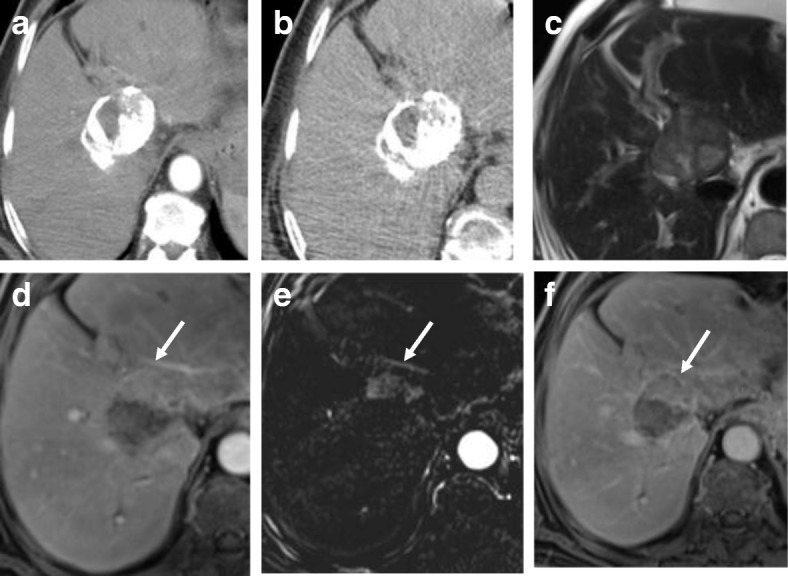
CT versus MRI after TACE. **a** Arterial phase CT shows heterogenous lipiodol condensation without appreciable enhancement. **b** Delayed phase CT shows heterogenous lipiodol condensation without appreciable washout. **c** T2-WI shows a heterogenous signal. **d** Enhanced T1-WI in the arterial phase shows non-enhancement in most of the lesion with faintly enhancing peripheral nodule (arrow). **e** Subtracted image confirms non-enhancement in most of the lesion with a faintly enhancing peripheral nodule (arrow). **f** Enhanced T1-WI in the delayed phase shows washout of the faintly enhancing peripheral nodule (arrow). Final diagnosis is LR-TR viable which is highlighted by MRI, not by CT

Hepatocellular carcinoma (HCC) constitutes a major cause of morbidity, mortality, and high health care cost in Egypt. In the past decade, there is a rapid rise in prevalence of HCC among Egyptian patients with hepatitis C virus infection which is almost a twofold increase [1]. HCC is an aggressive tumor constituting the third leading cancer-related deaths worldwide [2]. Treatment options for HCC are variable depending on stage of HCC at the time of diagnosis. Curative treatments are surgical including resection or liver transplant. Other curative options could be via locoregional therapies, e.g., thermal ablation (radiofrequency ablation [RFA] and microwave ablation) or chemotherapy-based conventional transarterial chemoembolization (TACE) as a bridge for liver transplant. Palliative treatments are transcatheter therapies including TACE, drug-eluting bead TACE (DEB-TACE), bland transarterial embolization (TAE), transarterial radioembolization (TARE), or systemic therapy. Such locoregional therapies are more popular in Egypt due to the delayed diagnosis, excessive cost of surgical options, presence of portal hypertension, and the non-availability of matching donor [3]. Assessing treatment response is crucial in therapeutic planning to evaluate the early need of repeated treatment [4]. This assessment could be performed by CT or MRI with no definitive evidence of superiority of MRI over CT in treatment response assessment. The choice of imaging modality in treatment response is chosen according to availability, institutional preference, and expertise. CT is widely used and faster than MRI, yet some pitfalls occur especially after lipiodol-based TACE due to beaming artifact of lipiodol hindering the proper evaluation of residual viable tumor [5] (Fig. 1). Also, the obvious contrast resolution of MRI especially with subtracted images helps in discriminating post-therapy changes from residual or recurrent tumor as well as detecting newly developed lesions [6]. Moreover, MRI is superior to CT in the surveillance for patients after treatment to minimize radiation exposures and hazards of contrast agents especially if young-aged [5, 7–12].

## Main structure

### Background of the available locoregional therapy

The available locoregional therapies for HCC in Egypt are thermal ablation (RFA and microwave ablation), TACE, DEB-TACE, TAE, TARE, and percutaneous ethanol injection. Percutaneous ethanol injection is regressing due to the availability of RFA. According to the Barcelona Clinic Liver Cancer classification, tailored therapeutic options could be used according to every case scenario via a multidisciplinary team, e.g., performance status, Child-Pugh score, tumor size, location of HCC and their number, presence of vascular invasion, portal hypertension, and extrahepatic spread [13].

In early-stage HCC, RFA is the best option with comparable results as surgical resection in terms of overall survival and disease-free survival [14]. In early-stage HCC, RFA shows complete response rates of 97% with 5-year survival rates of 68% [15]. RFA induces coagulative necrosis of the tumor by heat. However, the relation of vascular structures adjacent to tumor may induce heat-sink effect limiting the use of RFA due to the cooling effect by the adjacent vessel. Hence, the newer thermal ablative technique, microwave ablation, had emerged to overcome such problem. Moreover, it is more useful in larger tumors than RFA [16]. In intermediate-stage HCC, TACE is the best option either with conventional TACE or TACE with drug-eluting beads [17].

### MRI technique

The MR study should be done on high-field MR machine, either a 1.5-T or 3.0-T unit. Torso-phased array coils and breath-hold technique should be advocated. Parallel imaging should be used to improve SNR by decreasing acquisition time. 3.0-T MRI offers higher SNR than 1.5 T but more vulnerable to susceptibility artifacts [18–20]. Conventional anatomical MR imaging should include axial FSE T2-weighted imaging (T2-WI) with and without fat saturation with controlled breathing, axial gradient-recalled echo (GRE) DIXON T1-weighted imaging (T1-WI). Multiplanar imaging is optional. Diffusion-weighted imaging (DWI) is obtained by a free-breathing monopolar-3D Diagonal planar echo-pulse sequence (EPI) using parallel imaging. Multiple *b* values (at least two *b* values: 0 and 800 s/mm^2^) are used to generate ADC map and extract the ADC value. DWI has several limitations, e.g., poor SNR and susceptibility to several artifacts, including blurring, ghosting, and distortions especially for left lobe focal lesions. Also, the non-uniform fat suppression is one of the limitations of DWI on 3.0 T. Multiple solutions were offered to overcome these limitations, e.g., multichannel coils, strong gradients, high magnetic fields, breath-hold and ECG-gated techniques (if feasible), and advanced software [20–23]. Axial 3D fat-suppressed GRE T1-weighted imaging before and after dynamic injection of extracellular gadolinium-based contrast agents with an MR compatible pump injector was proposed. The dynamic study is performed with breath-hold in triphasic fashion: arterial phase (by bolus-tracking technique), portal venous phase (90 s), and delayed (equilibrium) at 3 min. Subtracted images mainly from the arterial phase dynamic study could be extrapolated to highlight subtle enhancing parts and to get rid of the signal from any hemorrhagic or fatty elements. Dynamic contrast-enhanced MR (DCE-MR) could be also performed.

### Treatment assessment criteria

The Response Evaluation Criteria in Solid Tumors (RECIST), incorporating unidimensional measurements, was addressed to evaluate change in tumor size after systemic treatments regardless of changes in the vascularity or necrosis of the tumor [24, 25].

Effective locoregional therapy for HCC aims to induce tissue necrosis, which occurs even before size change occurs. Moreover, after locoregional therapy, the treated HCCs may show increased size due to edema, hemorrhage, and necrosis [26]. Due to these limitations of RECIST, new assessment criteria by the European Association for the Study of the Liver (EASL) were considered. It relies on assessing the enhancing component of the tumor, incorporating bidimensional measurements, i.e., modified WHO bidimensional measurements [27, 28]. Then, modified RECIST (mRECIST) was introduced using the single largest diameter of the viable enhancing tumor during the arterial phase. Thereby, it is more practical for clinical use. According to EASL and the European Organisation for Research and Treatment of Cancer (EORTC), mRECIST criteria is the best used method after locoregional treatment for HCC on CT or MRI performed 1 month after therapy [29]. The mRECIST and EASL are good predictors of survival and for assessing anti-angiogenic effect of TACE [30–32] whereas there was no significant association between survival and RECIST 1.1 response [33].

Liver Imaging Reporting and Data Systems (LI-RADS) v2018 is recently addressing treatment response criteria. It offers imaging criteria for viable and nonviable HCC and introduces additional terms of nonevaluable tumors as well as equivocal viability [34]. The mRECIST addresses patient-level assessments according to changes in the sum of diameters of target lesions. However, mRECIST does not address the variable imaging features following different locoregional therapies [33]. The new LI-RADS treatment response algorithm offers a comprehensive approach for lesion-by-lesion assessment, using either contrast-enhanced CT or MRI. The viable tumor measurements obtained from the LI-RADS algorithm could be used as a patient-level response assessment via mRECIST. The viability defined by LI-RADS treatment response algorithm, not only hyperenhancing portions, was developed to improve patient outcomes. Also, LI-RADS facilitate communication with the treating physician via a common language. The limitations of LI-RADS include no treatment-specific algorithm available and no ancillary features applied in treatment response assessment. Further research and user feedback are required to validate these data from the LI-RADS treatment response algorithm [35].

### LI-RADS treatment response algorithm

#### LR-TR nonevaluable

This is mentioned if there is image degradation or omission.

#### LR-TR nonviable

This is mentioned when there is no appreciable lesional enhancement or treatment-specific enhancement.

#### LR-TR viable

If there is nodular, mass-like, or thick irregular tissue enhancement in or along the treated lesion with any of the following: arterial phase hyperenhancement OR washout appearance OR enhancement similar to pretreatment, then it is considered a viable tumor.

#### LR-TR equivocal

It is assigned when there is uncertainty if the lesion is nonviable or viable with atypical enhancement.

So, to sum up, first, the LI-RADS treatment response algorithm is applied and then the viable enhancing component is measured at the longest dimension through the enhancing area of the treated lesion, not traversing the non-enhancing area. Lastly, if there is uncertainty between the two categories, the one reflecting lower certainty is chosen, i.e., LR-TR equivocal [34].

## MRI appearance after locoregional treatment

### Thermal ablation

#### Lesion assessment

##### LR-TR nonevaluable

**Fig. 2:**
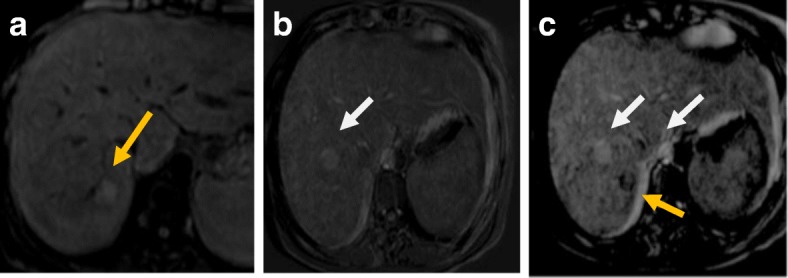
MRI after RF. **a** Pre-contrast T1-WI fat-suppressed image shows the ablated HFL at subsegment VII (yellow arrow) with its bright signal due to coagulative necrosis. **b** Subtracted arterial phase at a higher level shows slice mis-registration between the pre-contrast and post-contrast image and mirror image of the aorta (white arrow). **c** Subtracted arterial phase at the HFL shows slice mis-registration between the pre-contrast and post-contrast image and mirror image of the aorta (white arrow) with eccentric faint enhancement at the lesion (not consistent with the residual viable tumor). Final diagnosis is LR-TR nonevaluable due to image degradation

Proper evaluation could not be done due to image degradation (Fig. 2).

##### LR-TR nonviable

**Fig. 3:**
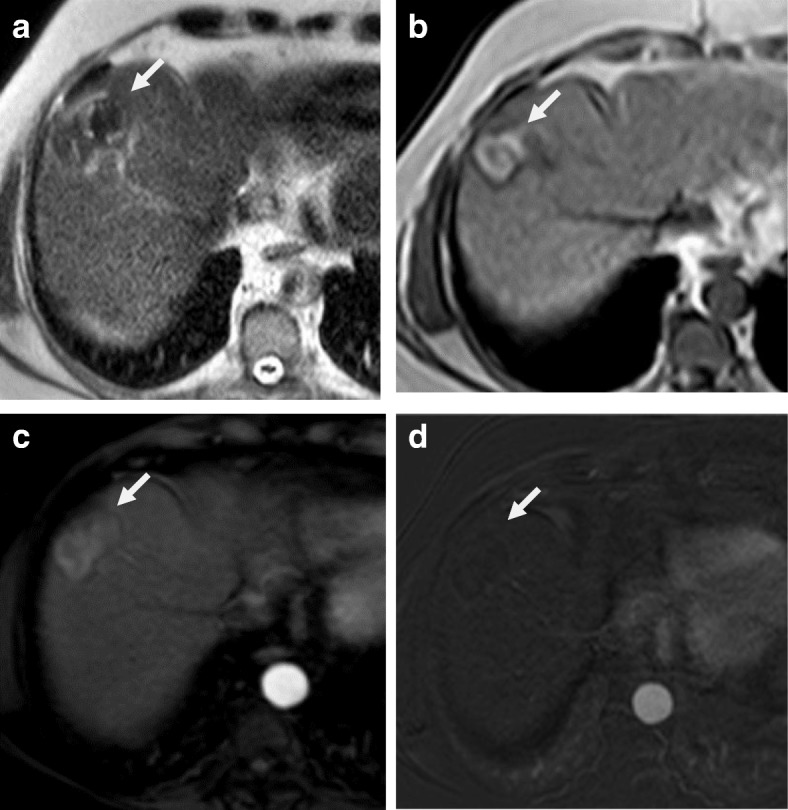
LR-TR nonviable after RFA. **a** T2-WI shows that the treated lesion (arrow) has a low signal lesion (arrow). **b** T1-WI before contrast shows a high signal of the lesion. **c** Enhanced T1-WI in the arterial phase shows a high signal of the lesion that can be misinterpreted as an arterial hyperenhancing focal lesion. **d** Subtracted image of the enhanced T1-WI from the non-enhanced T1-WI shows no appreciable enhancement

**Fig. 4:**
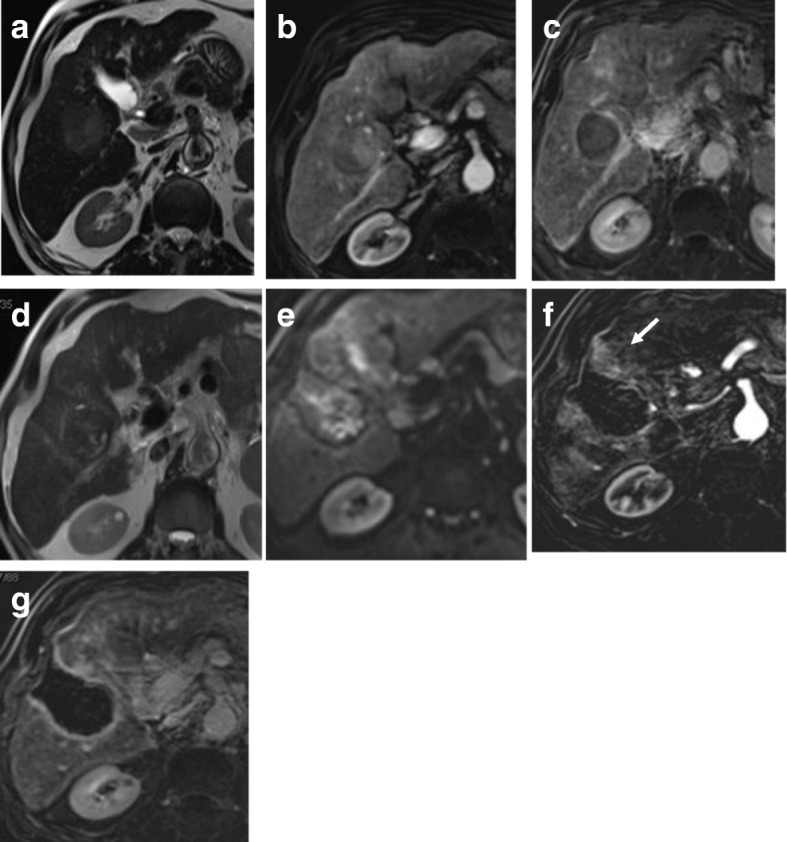
Hepatic focal lesion before (**a**–**c**) and after RFA (**d**–**g**). **a** T2-WI shows a well-defined HFL of an intermediate signal. **b** Enhanced T1-WI in the arterial phase shows early arterial heterogeneous enhancement. **c** Enhanced T1-WI in the delayed phase shows washout with delayed capsule enhancement. **d** After treatment, T2-WI shows a low signal with peripheral hyperintensity. **e** DWI shows central areas of restriction. **f** Subtracted T1-WI arterial phase shows non-enhancement of the lesions with faint peripheral enhancement (arrow). **g** Delayed T1-WI shows persistent peripheral enhancement. Final diagnosis is LR-TR nonviable with post-therapy changes

**Fig. 5:**
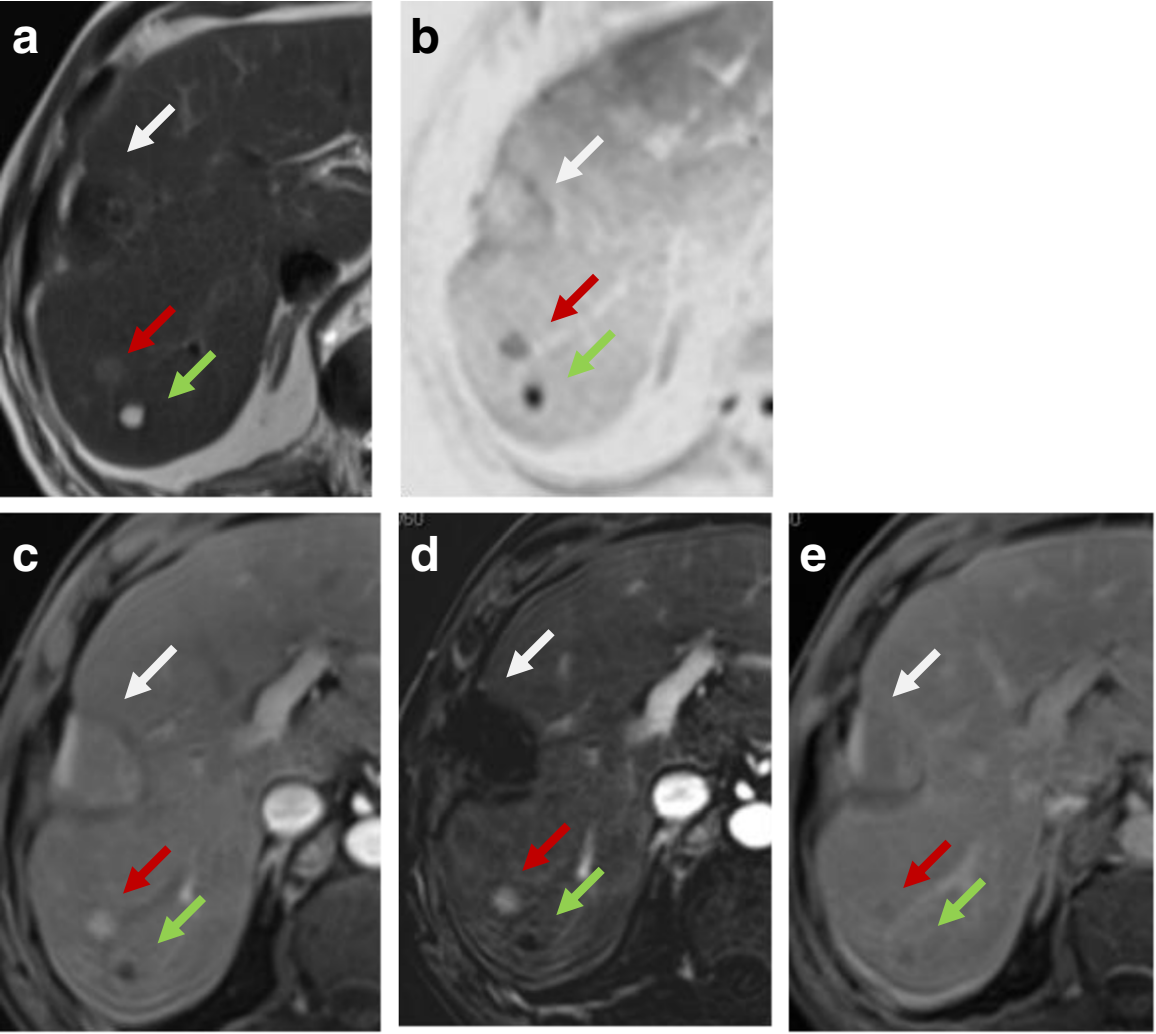
Hepatic focal lesion after RFA. **a** T2-WI shows a well-defined treated subcapsular focal lesion (white arrow). Two other smaller lesions are seen posteriorly; one is of an intermediate signal (red arrow) and the other is of a high signal (green arrow). **b** Inverse of DWI shows no restriction of the treated lesion (white arrow), yet shows restriction of the other two lesions. ADC (not shown) shows T2 shine through of the posterior lesion (green arrow) with true restriction of the anterior lesion (red arrow). **c** Enhanced T1-WI in the arterial phase shows a high signal of the treated lesion that can be misinterpreted as an arterial hyperenhancing focal lesion. Note the enhancement of the anterior lesion and non-enhancement of the posterior small lesions. **d** Subtracted image shows no appreciable enhancement of the treated lesion and posterior lesion, yet shows definite enhancement of the red-arrowed lesion. **e** Delayed T1-WI shows washout of the red-arrowed lesion. Final diagnosis is LR-TR nonviable, new small HCC, and small hepatic cyst

Thermal ablation induces coagulative necrosis. On T1-WI, this appears as a high signal or hyperintense peripheral rim while on T2-WI it appears as a low signal [36]. On the dynamic study, we prefer to use subtracted images to assess treatment response to nil any high signal within the lesions on the pre-contrast images facilitating discrimination between post-therapy changes and residual viable tumor [37]. On DWI, there is a hypointense central portion of the lesion while the periphery of the lesion shows hyperintensity with a lower ADC value than the surrounding hepatic parenchyma. This rim around the ablative zone is due to hyperemia and edema. The restricted diffusion of the edema occurs if it is a cytotoxic edema induced by the thermal stress. So, there is a challenge to discriminate post-treatment changes from residual tumors because both may cause restricted diffusion [38] (Figs. 3, 4, and 5).

#### Perilesional hepatic parenchyma signal alterations and enhancements

Sometimes, the adjacent hepatic parenchyma may show inflammatory changes. It shows a high signal on T2-WI with a low signal on an unenhanced T1-WI signal [39]. After contrast injection, this shows thin smooth rim enhancement [40]. This is known as transient hyperemia, and it is an expected finding in initial imaging after thermal ablation [41]. These immediate changes will disappear on subsequent imaging. This must be differentiated from a residual viable tumor which appears as nodular or thick peripheral enhancement [42–44] (Fig. 4).

##### LR-TR equivocal

If there is a suspicious small zone of enhancement, then short-term follow-up after 3 months would be recommended [45].

##### LR-TR viable

**Fig. 6:**
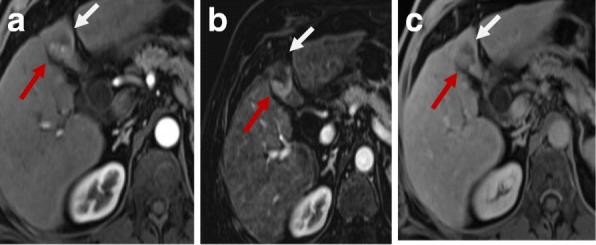
Hepatic focal lesion after RFA. **a** Enhanced T1-WI in the arterial phase shows early arterial peripheral nodular enhancement (red arrow). **b** Subtracted T1-WI arterial phase shows true enhancement of the nodule (red arrow). **c** Enhanced T1-WI in the delayed phase shows washout with capsule enhancement (red arrow). Overall post-treatment assessment is LR-TR viable

If there is early enhancement and delayed washout, this is considered as a residual viable tumor [44] (Fig. 6).

### Complications

Complications of thermal ablation are minimal, and these include injury to blood vessels with formation of arteriovenous shunt. Subsequently, a wedge area of enhancement will be seen without washout. Other complications could be injury of bile ducts with biloma formation or injury to adjacent organs, e.g., the gall bladder, kidney, and bowel [46]. Infection and abscess formation is reported in the literature yet not common. Needle track seeding could also happen yet very rarely [40, 47].

### Transarterial chemoembolization

#### Lesion assessment

It is preferred to perform MR rather than CT in lipiodol-based embolization because the beaming artifact of lipiodol on CT hinders the proper assessment of a residual viable tumor [5], yet complete lipiodol uptake is strongly associated with a complete response at pathology and can be used as an additional criterion.

##### LR-TR nonviable

A well-treated necrotic HCC shows variable signal intensity on unenhanced T1-WI and T2-WI due to liquefactive necrosis, hemorrhage, and inflammation [48]. A well-treated lesion shows no enhancement on dynamic study [49, 50].

#### Perilesional hepatic parenchyma

As in thermal ablation, transient hyperemia is seen at the periphery of the treated lesion.

##### LR-TR viable

**Fig. 7:**
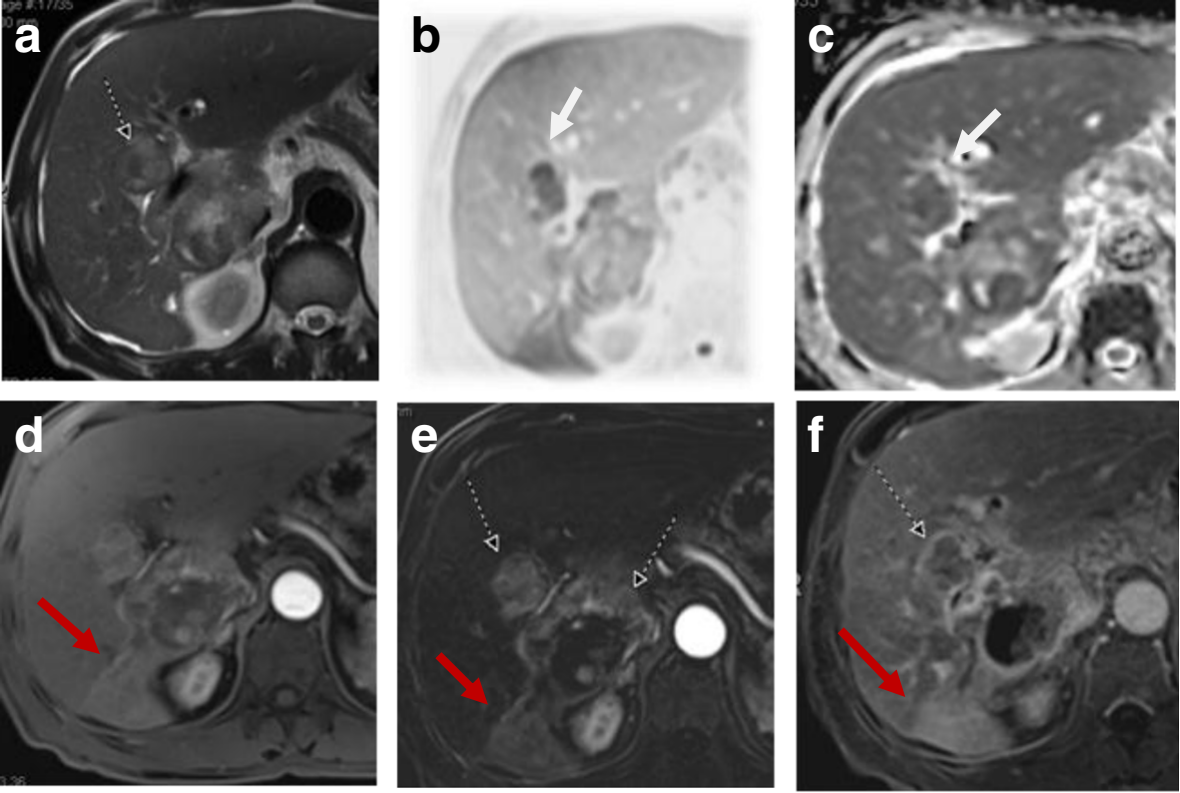
Hepatic focal lesion after TACE. **a** T2-WI shows two well-defined HFLs of an intermediate to high signal. **b**, **c** DWI and ADC show central areas of true restriction for the smaller lesion (arrow) and heterogeneous restriction for the larger one. **d** Enhanced T1-WI in the arterial phase shows early arterial heterogeneous enhancement. **e** Subtracted T1-WI arterial phase shows true enhancement of the lesions (dashed arrows). There is a wedge area of enhancement as well (red arrow). **f** Enhanced T1-WI in the delayed phase shows washout with capsule enhancement (dashed arrow). The wedge enhancement shows delayed T1-WI persistent enhancement (red arrow). Overall post-treatment assessment is LR-TR viable with post-therapy changes

The presence of peripheral nodular enhancement with washout is an indication of the presence of a viable tumor either residual or re-growth [51] (Fig. 7).

### Complications

**Fig. 8:**
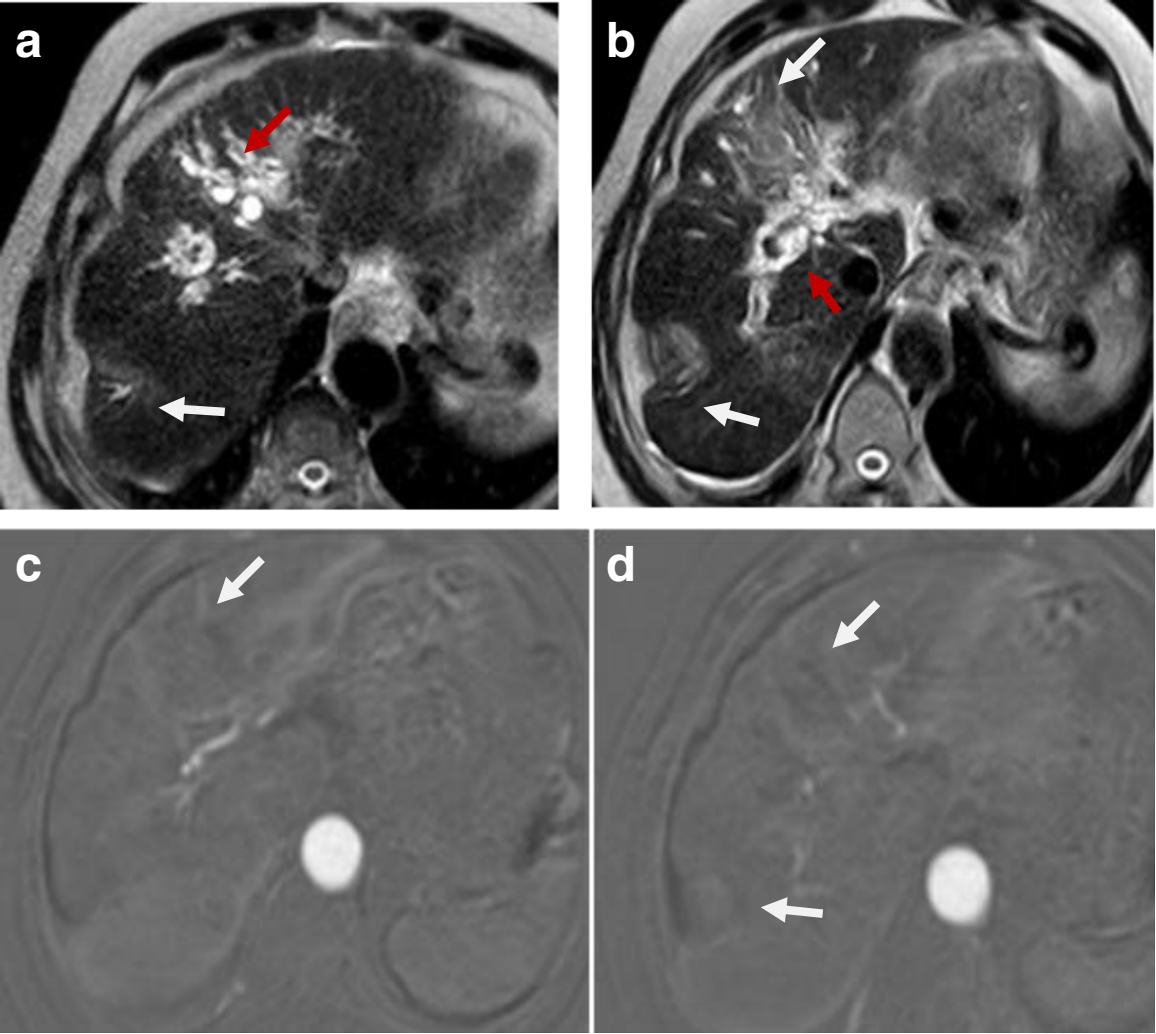
Hepatic focal lesions after TACE. **a**, **b** T2-WI shows two well-defined HFLs of an intermediate to high signal (white arrows) with central biliary dilatation (red arrow). **c**, **d** Subtracted T1-WI arterial phase shows no enhancement of the lesions. Overall post-treatment assessment is LR-TR nonviable with post-therapy biliary dilatation

**Fig. 9:**
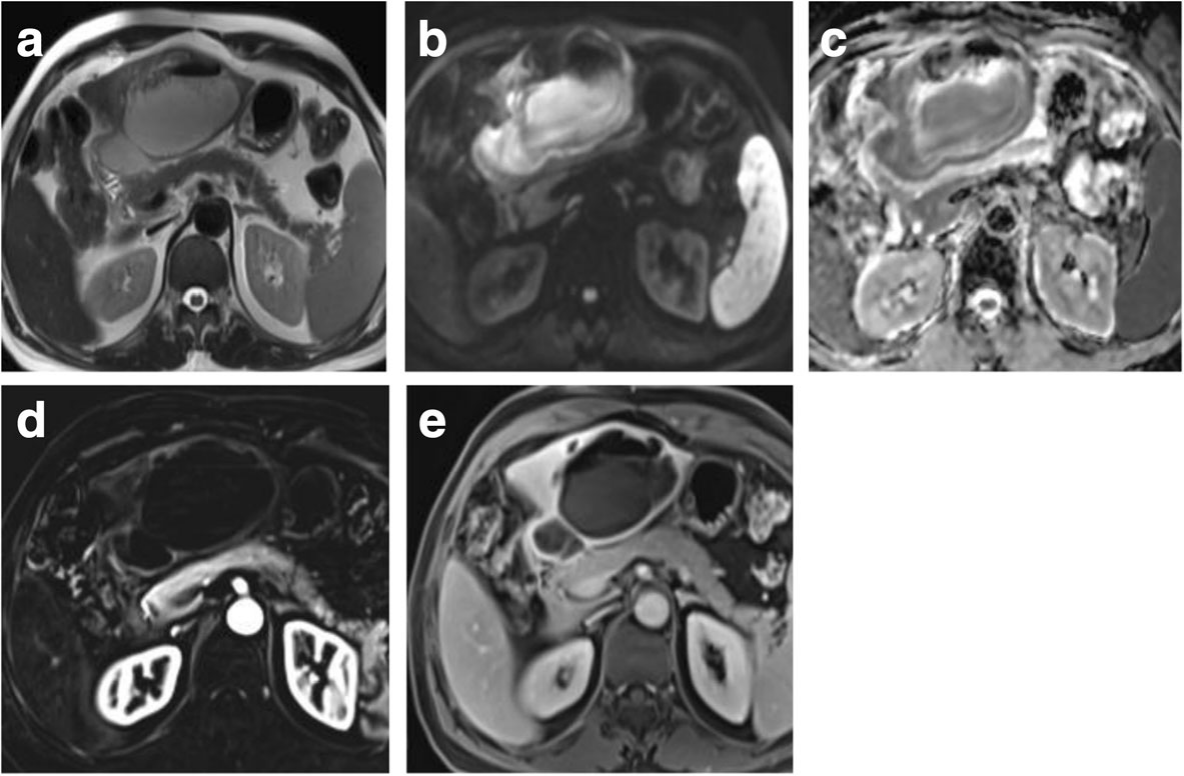
Hepatic focal lesion after TACE. **a** T2-WI shows a well-defined cystic lesion at the treated focal lesion with air/fluid level. **b**, **c** DWI and ADC show central areas of true restriction. **d** Subtracted T1-WI arterial phase shows peripheral smooth wall enhancement of the lesion. **e** Post-contrast T1-WI delayed phase shows peripheral smooth wall enhancement of the lesion. Overall post-treatment assessment is LR-TR nonviable with post-therapy abscess formation

Arterioportal shunts could occur after treatment due to injury of small hepatic arteries. Other complications include abscess formation or biloma [52]. Embolization of arteries other than the selected artery for the HCC can cause injury to the related organ and leak of the chemotherapy agent to the systemic circulation [53] (Figs. 8 and 9).

### Radioembolization

**Fig. 10:**
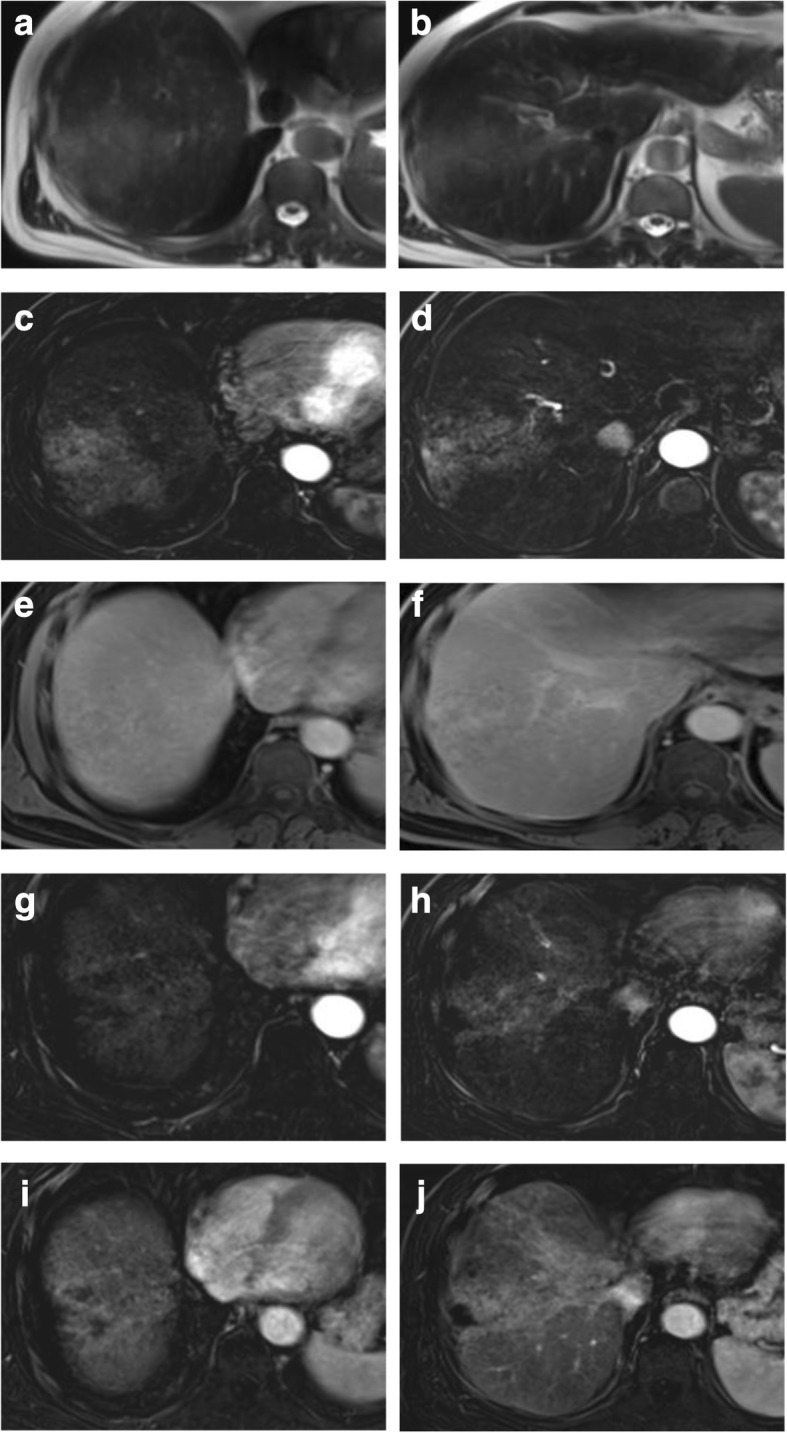
Hepatic focal lesion before and after TARE. **a**, **b** T2-WI shows an ill-defined HFL of an intermediate signal. **c**, **d** Subtracted enhanced T1-WI in the arterial phase shows early arterial heterogeneous enhancement. **e**, **f** Enhanced T1-WI in the delayed phase shows heterogenous washout. **g**, **h** After treatment, subtracted T1-WI arterial phase shows non-enhancement of the upper portion of the lesion with faint enhancement lower portion. **i**, **j** Delayed T1-WI shows delayed and persistent enhancement of the lesion due to massive fibrosis. Final diagnosis is LR-TR nonviable with post-therapy changes

**Fig. 11:**
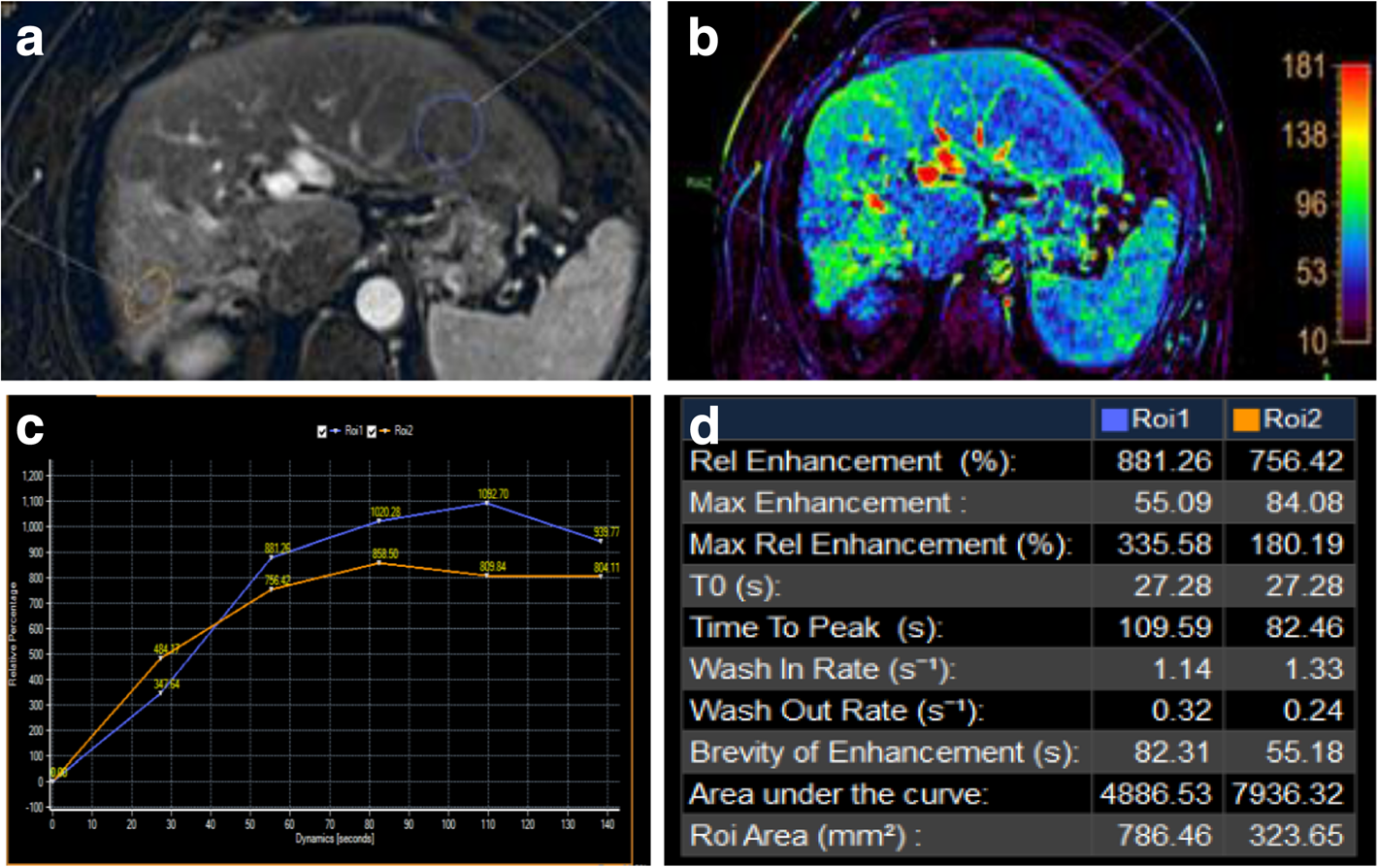
Hepatic focal lesion after TARE. **a** Subtracted enhanced T1-WI in the arterial phase shows faint enhancement of the treated right lobe (ROI 2 “orange”) compared to untreated hepatic parenchyma (ROI 1 “blue”). **b** T1 perfusion map (max. relative enhancement) shows mild increased blood flow to the treated right lobe. **c** Time-intensity curves on the treated right lobe (orange) and untreated left lobe (blue) show an uprising slope and followed by a plateau for the lesion due to fibrosis. **d** Semi-quantitative data for both ROIs show decreased max. relative enhancement, time to peak, wash in rate, and wash out rate of the treated hepatic parenchyma compared to the rest of the hepatic parenchyma. Final diagnosis is LR-TR nonviable with post-therapy changes

It induces extensive inflammation of the hepatic parenchyma that can be misdiagnosed as a viable lesion [54, 55]. The absence of contrast washout in delayed images and the gradual decreased enhancement as well as shrinkage of the lesion over time are a good sign for a well-treated focal lesion [56] (Figs. 10 and 11).

### Functional assessment

Functional imaging is the use of advanced tools in assessing cellularity and vascularity of a hepatic focal lesion. They could give qualitative and quantitative data about tumor response even before morphological changes. These include diffusion and perfusion imaging [57].

### Diffusion-weighted imaging (DWI)

DWI is a promising functional biomarker tool which assesses cellularity of the tissues depending on motion of water molecules at the extracellular/extravascular space. So, the highly cellular tissues show restricted motion of water molecules, yet the less cellular tissues allow free motion of water molecules [58, 59]. Using high *b* values (800–1000 s/mm^2^) allows suppression of background signals from the normal liver parenchyma and results in increased contrast between the normal liver and lesions, facilitating the detection of hepatic focal lesions [60]. DWI enables the detection of small lesions around vessels and in the periphery of the liver which is sometimes challenging to be detected on T2-WI [61, 62]. DWI is also valuable especially when contrast media is contraindicated [63–65].

**Fig. 12:**
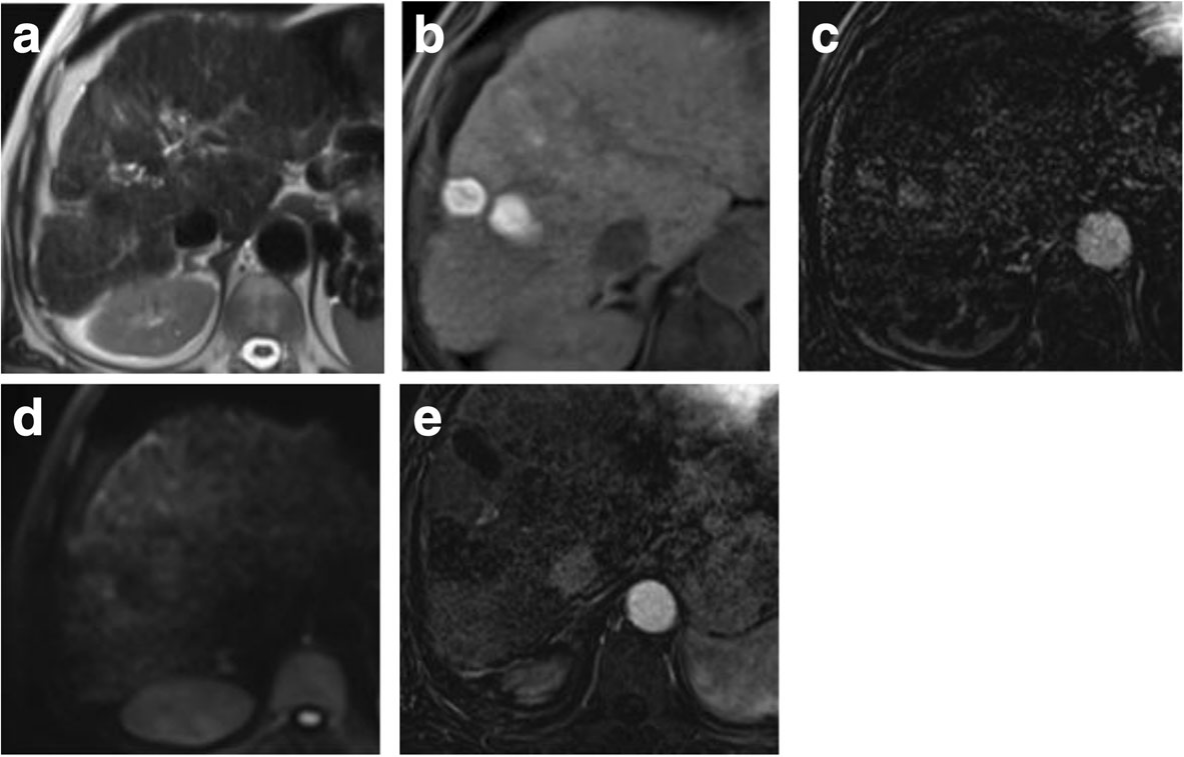
Two hepatic focal lesions after old RFA. **a** T2-WI shows a hypointense signal of the treated focal lesions. **b** Pre-contrast T1 fat-suppressed image shows the ablated HFLs with their bright signal due to coagulative necrosis. **c** Subtracted T1-WI arterial phase shows newly developed faint enhancement of the lesions. **d** DWI shows no true restriction corresponding to the enhancement. **e** Subtracted T1-WI arterial phase after a 3-month interval shows no enhancement of the lesions. Initial post-treatment assessment by LI-RADS v2018 is LR-TR equivocal that proved to be nonviable after a short-interval follow-up. DWI was consistent with LR-TR nonviable from the start with no restriction initially and on the follow-up (not shown)

DWI is helpful in lesion characterization of a hepatic focal lesion (HFL) in a cirrhotic liver where a combination of restricted diffusion with arterial hyperenhancement is more likely to be HCC [66, 67]. Also, it is helpful in assessing tumoral versus bland thrombosis of the portal vein [68]. DWI and ADC show an additive value in detection of viable malignant hepatic focal lesions following locoregional therapy [58, 69, 70] (Fig. 12).

### Post-TACE assessment

Kamel et al. [71] showed that there are different ADC values between necrotic portions compared to the viable tumors after TACE. They stated that the significant difference will be at 1–2 weeks after TACE and is correlated to enhancing components, which in turn also shows maximum difference from normal hepatic parenchyma after 1–2 weeks. Mannelli et al. [49] found that there was an insignificant difference between ADC values and subtracted images. The increase in the ADC value is an indication of good response to treatment reflecting less cellular packing [72]. This was also agreed with a study done by Chapiro et al. [73]. Bonekamp et al. [74] had investigated volumetric ADC changes after TACE at 1-month post-therapy and correlated with 6-month RECIST and mRECIST response. Volumetric ADC values are increased with objective response by mRECIST at 6 months with a sensitivity of 88.4% and specificity of 78.6% (*p* = 0.001). Comparable results were obtained by using RECIST response criteria, yet with lower sensitivity and specificity. Sahin et al. [75] observed that an absolute increase in ADC values can differentiate viable/contrast-enhancing (1.42 ± 0.25 × 10^–3^ mm^2^/s) and necrotic/non-enhancing (2.22 ± 0.31 × 10^–3^ mm^2^/s; *p* < 0.001) tumor areas when compared to contrast enhancement patterns 6–8 weeks after TACE. Yuan et al. [76] found ADC threshold of 1.84 × 10^− 3^ mm^2^/s, which can differentiate necrotic from non-necrotic portions with 92.3% sensitivity and 100% specificity.

### Post-TARE and radiation therapy (RT)

Kokabi et al. [77] demonstrated that absolute ADC value changes were an imaging biomarker for a prompt response assessment in patients with HCC and portal vein thrombosis. Rhee et al. [78] showed that objective mRECIST responders after 3 months had a significantly greater mean ADC after 1 month than non-responders. Also, percent of ADC increase was also significantly higher in responders compared to non-responders at 3-month post-TARE. They found that an increase of > 30% in the ADC value after 3 months predicts treatment response with 90% sensitivity and 100% specificity. Park et al. [79] found that DWI improves detection of a viable tumor after radiation therapy (RT) for HCC. Yu et al. [80] found that the local progression-free survival is related to the change in the ADC value before and after RT for HCC. Hence, if there is a contraindication for contrast agents, the ADC value and RECIST may substitute for mRECIST.

### Post-thermal ablation

Post-thermal ablation induces hemorrhage which can mask the post-treatment expected ADC increase limiting the use of DWI in treatment response assessment [81, 82]. Schraml et al. [83] showed that the ADC value of signal alterations adjacent to the ablation zone may help in identification of local tumor progression and non-tumoral post-treatment changes.

### ADC value assessment

ADC is recently investigated as a pre-treatment biomarker to predict treatment response for TACE [49]. There is conflicting data in the literature about which tumors with high or low ADC values are good responders to TACE [49, 84, 85]. These conflicting data of DWI and ADC could be due to the use of different number of *b* values in each study and complexity of lesion components of vascularity, hemorrhage, and necrosis [86, 87]. Padhani et al. [88] suggested that lesions with a higher vascularity exhibit a more restricted diffusion with lower ADC values. This could explain the better outcomes in lesions with lower pretreatment ADC values [84, 89–91]. Necrotic lesions show higher ADC values, and it is a sign of tumor aggressiveness. Necrotic lesions are poorly perfused and, therefore, could explain the poor outcomes in lesions with higher pretreatment ADC values [49, 84, 89, 91].

DWI has demonstrated a significant lower sensitivity and high specificity for tumor recurrence detection when compared to DCE-MRI. Therefore, DWI has no role in detection of recurrence [92].

### Perfusion

**Fig. 13:**
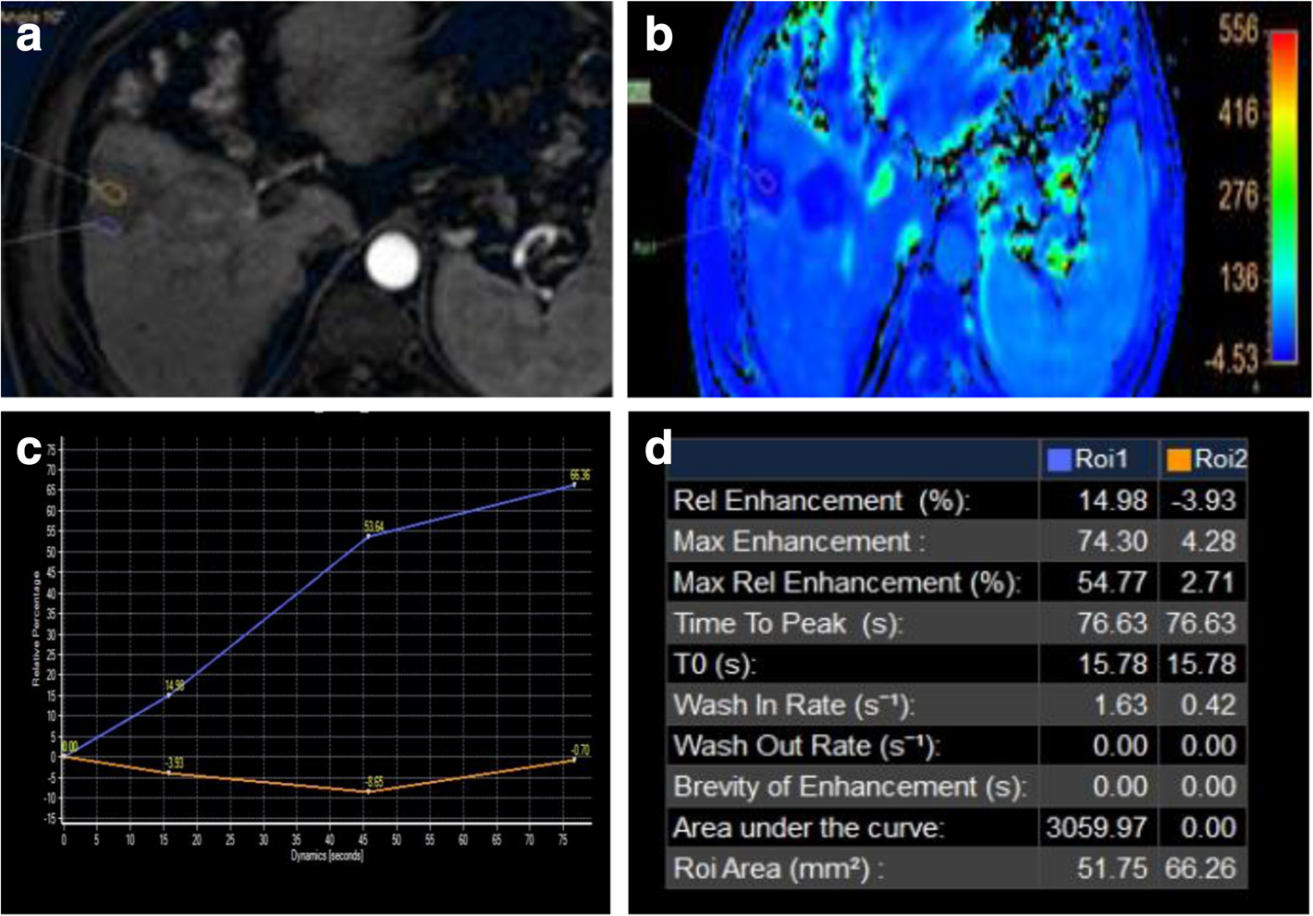
Two hepatic focal lesions after RFA. **a** Subtracted enhanced T1-WI in the arterial phase shows faint enhancement of the periphery of the treated HFLs (ROI 1 “blue”) compared to the center of HFLs (ROI 2 “orange”). **b** T1 perfusion map (max. relative enhancement) shows mild increased blood flow peripherally. **c** Time-intensity curves on the periphery of the treated HFLs (blue) and their center (orange) show an uprising slope due to inflammation. **d** Semi-quantitative data for both ROIs show significant decreased max. relative enhancement of the center of treated HFLs compared to the periphery. Final diagnosis is LR-TR nonviable with post-therapy changes

Dynamic contrast-enhanced MRI (DCE-MRI) with IV contrast injection uses high-temporal images to assess changes in MR signal intensity (SI) over time. This has been used to detect liver fibrosis and cirrhosis and to assess tumor angiogenesis [93–95]. DCE-MRI can quantify vascularity of tumors and their response to angiogenic drugs especially for metastatic hepatic focal lesions and to sorafenib treatment. The obtained values via tracer kinetic modeling could be used in future therapeutic monitoring [96–102]. There are few prior studies for DCE-MRI assessing HCC lesions especially after locoregional therapy [98, 103, 104]. Taouli et al. [99] have found differences in perfusion parameters between untreated HCCs and the background liver with higher arterial hepatic blood flow and arterial fraction, lower portal venous hepatic blood flow for HCCs, and no difference in distribution volume and mean transient time (MTT) (Fig. 13). Moreover, they found a decrease in arterial fraction, arterial hepatic blood flow, and distribution volume and an increase in portal venous hepatic blood flow after TACE. Sahani et al. [105] quantified perfusion on dynamic CT in patients (*n* = 30) with untreated HCC. They found that HCCs had higher blood flow, blood volume, and permeability–surface area product compared with the liver parenchyma, but MTT was lower in tumors. Abdullah et al. [94] assessed perfusion parameters in HCCs (*n* = 26) and colorectal metastases (*n* = 24). They found significantly higher arterial hepatic blood flow, portal venous hepatic blood flow, total hepatic blood flow, and distribution volume in HCCs compared with metastases. Also, they found significantly lower MTT in HCCs in comparison to metastases. They also found that HCCs showed higher portal venous hepatic blood flow than arterial hepatic blood flow. Dynamic CT has a direct linear relation between signal enhancement and iodine concentration making it superior to DCE-MRI. Yet, perfusion CT has a disadvantage of radiation exposure, especially for follow-up studies. Also, multiparametric tools of MRI with DCE-MRI and diffusion-weighted MRI have the advantage over dynamic CT in better characterization of angiogenic activity and treatment response of HCC to TACE [99].

### Recent advances in MRI

#### Intravoxel incoherent motion (IVIM)

IVIM is a novel MR technique which has been introduced to study both diffusion and perfusion effects on masses without the need of intravenous contrast injections and is specifically helpful for patients with renal impairment, with contrast allergy, or with fear of long-term effects of gadolinium deposition. This technique can separate pure diffusion characteristics (D) from pseudo-diffusion caused by microscopic circulation in tissues, with also calculation of perfusion characteristics (pseudo-diffusion coefficient D*) and its proportion (perfusion fraction fp) [106].

**Fig. 14:**
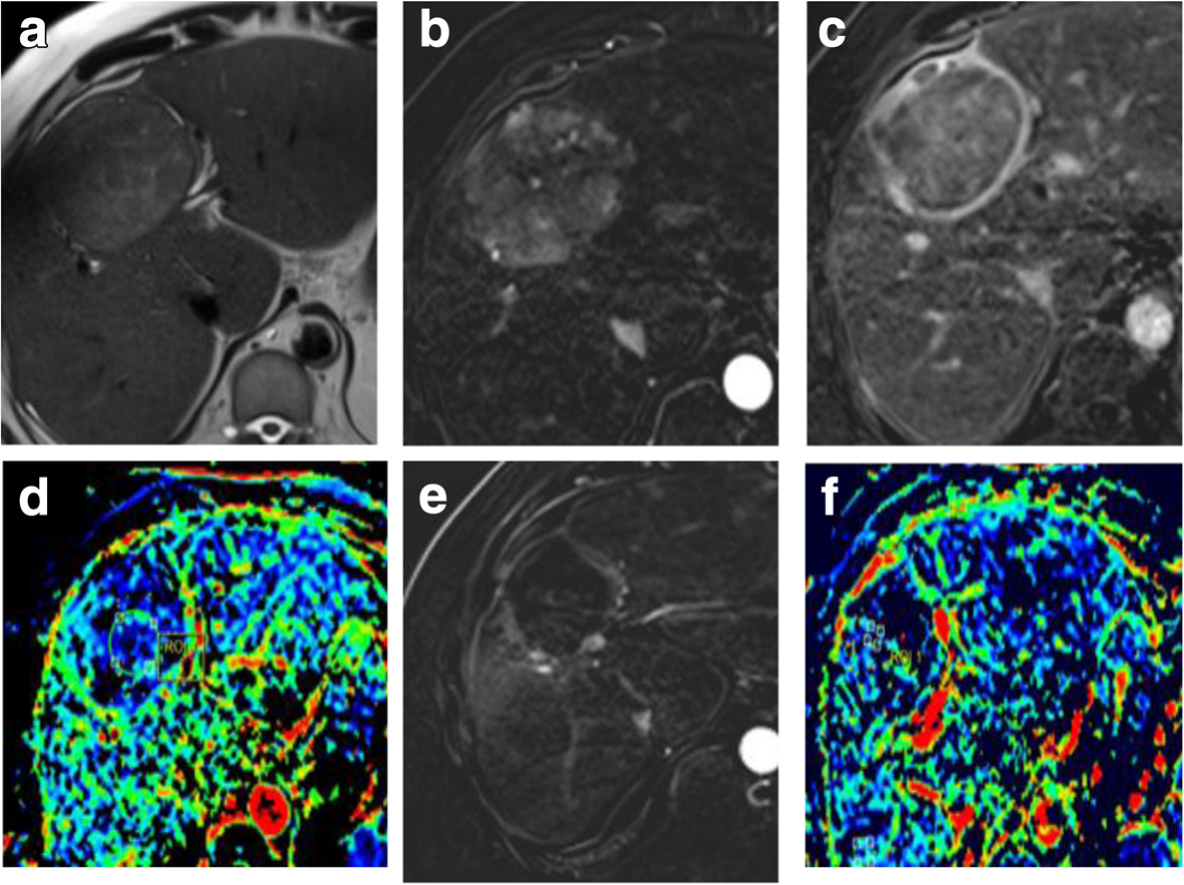
Hepatic focal lesion before and after TACE with IVIM assessment. **a** T2-WI shows a well-defined HFL of an intermediate signal. **b** Subtracted enhanced T1-WI in the arterial phase shows early arterial heterogeneous enhancement. **c** Enhanced T1-WI in the delayed phase shows washout with delayed capsule enhancement. **d** F map of IVIM shows a high *F* value due to increased microcirculation. **e** Post-treatment subtracted T1-WI arterial phase shows non-enhancement of the lesion. **f** Post-treatment F map of IVIM shows a reduced *F* value due to tumor necrosis. Final diagnosis is LR-TR nonviable

Woo et al. [107] and Mürtz et al. [108] found a significant correlation between IVIM-derived parameter (fp) and percent of arterial enhancement whether in the initial diagnosis of HCC or after the locoregional therapy. Hence, it could be used after locoregional therapy to distinguish responders from non-responders especially if there is contraindication for contrast administration (Fig. 14).

Regarding D and ADC data, D-derived IVIM parameter was better than the ADC value to assess tumor necrosis after locoregional therapy for HCC. This higher accuracy of D-derived IVIM parameter can be explained by the fact that ADC is a nonspecific value that contains combined information on tissue cellularity (D) and perfusion (microcirculation), causing opposite effects on the measurement of ADC, resulting in decreased sensitivity and specificity. However, D-derived IVIM parameter eliminates this perfusion (microcirculation) contaminated ADC values, allowing pure measurements of tissue cellularity [108, 109]. So IVIM is a promising tool for hepatic focal lesion assessments before and after locoregional therapy especially without the need for contrast injection.

## Summary

The LI-RADS v2018 is now the cornerstone in daily practice for evaluating treatment response of HCC after RFA and TACE, based on enhancement criteria of the treated focal lesion which is better correlated with subtracted images to omit post-treatment changes of coagulative necrosis and hemorrhage, because it is an easy and accurate method. However, it is more challenging to rely on this criterion alone especially after TARE due to complexity of post-treatment changes. Yet, the DWI/ADC value can help in the fine tuning of decision making in challenging equivocal cases especially if there is mismatch between diffusion restriction and enhancement to avoid unnecessary repeated treatment. Conventional MRI could not predict tumor grade, aggressiveness, angiogenesis, and hypoxia. Here comes the role of non-invasive MRI functional imaging including DWI, perfusion-weighted imaging, and the novel IVIM to be used as biomarkers to assess early treatment response of HCC to locoregional therapy especially after TACE and TARE. These functional data are better combined with morphological data to improve the diagnostic and prognostic criteria for post-locoregional treatment assessment of HCC. Ultimately, many studies are needed to validate a non-invasive algorithm based on multiparametric MRI to predict response of HCC and minimize the variability of the quantitative MRI metrics.

## Abbreviations

D*: Pseudo-diffusion coefficient
fp: Perfusion fraction
D: Pure diffusion characteristics
DEB-TACE: Drug-eluting bead TACE
EASL: European Association for the Study of the Liver
EORTC: European Organisation for Research and Treatment of Cancer
HCC: Hepatocellular carcinoma
HFL: Hepatic focal lesion
IVIM: Intravoxel incoherent motion
LI-RADS: Liver Imaging Reporting and Data System
mRECIST: Modified RECIST
RECIST: Response Evaluation Criteria in Solid Tumors
RFA: Radiofrequency ablation
SNR: Signal-to-noise ratio
TACE: Chemotherapy-based conventional transarterial chemoembolization
TAE: Bland transarterial embolization
TARE: Transarterial radioembolization
